# How to Best Convey Information About Intensive/Comfort Care to the Family Members of Premature Infants to Enable Unbiased Perinatal Decisions

**DOI:** 10.3389/fped.2018.00348

**Published:** 2018-11-16

**Authors:** Jingjing Gong, Wei Xiao, Hongyan Gao, Wei Wei, Weiwei Zhang, Jing Lv, Lijun Xiao, Lida Duan, Yan Zhang, Hongyun Liu, Yonghua Huang

**Affiliations:** ^1^Department of Neurology, PLA Army General Hospital, Beijing, China; ^2^Department of Medical Psychology, Air Force Medical University, Xi'an, China; ^3^Department of Medical Administration, PLA Army General Hospital, Beijing, China; ^4^Department of Psychology, PLA General Hospital, Beijing, China; ^5^Department of Paediatrics, PLA Army General Hospital, Beijing, China; ^6^Department of Medical Administration, PLA Zhurihe Base Hospital, Zhurihe, China; ^7^Center of Psychology, Air Force Aviation Medicine Research Institute, Beijing, China; ^8^School of Psychology, Beijing Normal University, Beijing, China

**Keywords:** intensive care, perinatal decision making, framing effect, latent class analysis, decision model

## Abstract

**Background:** As the infant's best interests are determined through the perinatal decisions of family members and physicians, it is important to understand the factors that affect such decisions. This paper investigated the separate and combined effects of various factors related to perinatal decision making and sought to determine the best way to convey information in an unbiased manner to family members.

**Methods:** In total, 613 participants were consecutively recruited. Each participant completed a series of surveys. All responses to four items were examined via a latent class analysis (LCA) to identify subgroups of participants with similar preferences for intensive care (IC) and comfort care (CC) regarding their potentially premature infant. Multiple logistic regression analyses were applied to identify the sociodemographic predictors for the classification of participants into different subgroups.

**Results:** χ^2^-tests indicated that perinatal decision making for Item 2 was influenced by framing information, whereas decision making wasn't affected by presentation modes. Furthermore, the endorsement rates of IC significantly decreased with the information increased from brief to detailed, regardless of framing or presentation mode. The LCA indicated that a 3-subgroup model, which included the IC, CC, and variation subgroups, was optimal. Logistic regression analyses demonstrated that, compared with the IC subgroup, negative framing, higher education, parenthood, and poor numeracy predicted participants' preferences for CC. Meanwhile, worrying about physical or mental disabilities predicted preferences for CC and sensitivity to the amount of information provided regarding treatment options (variation subgroup).

**Conclusions:** Perinatal decision making is affected by many factors, suggesting that more detailed information, improved understandability of numerical data, and a neutral tone of voice regarding therapeutic outcomes would be helpful for the families of premature infants to make unbiased decisions. Our findings should be replicated in future research.

## Introduction

In neonatology, the treatment of extremely premature infants (e.g., 23 weeks or less) depends on the attitudes of their family members and pediatricians regarding intensive care (IC) and comfort care (CC). Extremely preterm infants are referred to an IC unit, where many machines are employed to assist with survival (e.g., regarding breathing and heart rate). In contrast, infants under CC are simply kept warm and comfortable by doctors and nurses until they die on their own (1). Much of the literature concerning treatment decision making in neonatology has focused on clinician behavior, and little research has examined the parental decision-making process (1). Because the infant's best interests are determined through the perinatal decisions of family members and physicians, especially for extremely premature infants at 23 weeks or less, it is important to understand the factors that affect such decisions.

The framing effect can influence parental perinatal decision making (1, 2) (e.g., subjects react differently to IC vs. CC options for extremely premature infants depending on whether the prognosis is presented as survival or death), whereas the degree of detail provided about the options (CC or IC) does not (3). However, the degree of detail significantly influences participants' preferences for end-of-life palliative care for adults (4). Previous studies exploring whether the framing effect, the mode of presentation and the degree of the detail concerning treatment options are operative in prenatal decision making in cases of extreme prematurity have rarely been reported. Using latent class analysis (LCA), which is similar to cluster analysis (5), we investigated the separate and combined effects of the various aforementioned factors concerning prenatal decision making to guide physicians on how to best convey information about IC and CC in an unbiased manner to family members of premature infants.

## Materials and methods

### Participants

The present study was originally designed to be completed by pregnant women and their family members, especially those with the prospect of delivering an extremely preterm infant. However, the local ethics committee suggested that we perform a preliminary study examining additional inpatients and their relatives. As such, the current study was subsumed with other medical decision-making studies conducted at the Department of Neurology. A total of 613 consecutive inpatients from China without physical or mental disabilities or next of kin were recruited. The general participant eligibility criteria included (1) a modified Rankin Scale score of 0–1; (2) an age ≥18 years old; (3) Mini-Mental State Examination (Chinese revised version) scores of either >20 (for those with ≤6 years of education) or >24 (for those with >6 years of education); (4) the ability to read and complete the questionnaires; and (5) an absence of severe mental disorders. Participants who failed to complete the assessment were excluded, and 572 (93.31%) of the 613 participants completed the surveys (330 inpatients and 242 relatives). Their detailed sociodemographic data are shown in Supplementary Table S1.

The Academic Committee of the Army General Hospital approved this study. All methods were performed in accordance with the approved guidelines. Written informed consent was obtained from all participants.

### Study design

This survey, which was administered as part of a broader study of medical decision making, depicted scenarios of threatened delivery at a gestational age of 23 weeks (1). Inpatients and their kin whose infant prognosis was framed as survival data were randomly assigned to the positive framing group (Pos), whereas those whose infant prognosis was framed as mortality data were assigned to the negative framing group (Neg). Each participant received (1) a brief description of the delivery, IC, and CC, followed by Items 1 (the outcomes of IC and CC presented as small-base-specific figures; SF) and 2 (the outcomes of IC and CC presented as large-base-specific figures; LF; Table 1); (2) a detailed description of the delivery, IC, and CC (1) (Appendix 1); and (3) the same items as Items 1 and 2 (but renamed Items 3 and 4; Table 1). A series of surveys related to sociodemographics as well as health and emotion states were inserted into the middle of the four items, and a survey of participants' personal attitudes toward life (1) was included at the end.

**Table 1:** Content of the items across the different presentation modes and framing types.

**Information of background and treatment**	**Item no**.	**Modes of presentation**	**Frame type**	**Contents of items**
**BRIEF INFORMATION**				
	1	SF (100 infants as reference)	Negative	IC: Out of 100 infants, 75 will die. Of those who will not die, 10 will suffer from severe developmental disabilities. CC: Out of 100 infants, all infants will die (Which treatment do you prefer?).
			Positive	IC: Out of 100 infants, 25 will survive. Of those who survive, 15 out of 25 will not suffer from severe developmental disabilities. CC: Out of 100 infants, no infants will survive (Which treatment do you prefer?).
	2	LF (77,000 infants as reference)	Negative	IC: Out of 77,000 infants, 57,750 will die. Of those who will not die, 770 will suffer from severe developmental disabilities. CC: Out of 77,000 infants, all infants will die (Which treatment do you prefer?).
			Positive	IC: Out of 77,000 infants, 19,250 will survive. Of those who survive, 11,550 will not suffer from severe developmental disabilities. CC: Out of 77,000 infants, no infants will survive (Which treatment do you prefer?).
**DETAILED INFORMATION**				
	3	SF (100 infants as reference)	Negative	See Item 1
			Positive	See Item 1
	4	LF (77,000 infants as reference)	Negative	See Item 2
			Positive	See Item 2

### Materials

The assessments included instructions, a numeracy scale (6), different ways to present information about IC and CC (Table 1), sociodemographics, health and attitude questionnaires, and the SCL-90-R (7) (Appendices 2–6).

### Statistical analyses

χ^2^-tests were employed to explore the differences in the endorsement of IC/CC among individuals who received different sets of background information (brief or detailed) and disparate presentation modes (SF or LF). Because we compared four different groups repeatedly, the significance level was changed from α (0.05) to α′ (0.007) based on the partitioning of the χ^2^ method (α′ = α/[k^*^{k – 1}/2 + 1], K = 4) to reduce the error I. Regardless of the framing scenario, all participants' responses to the four items were analyzed via LCA performed using Mplus 7.0 (8) to identify different subgroups of participants (i.e., latent classes) according to their response patterns to IC/CC (9, 10). In other words, participants with similar response patterns to the four items were classified into the homogeneous subgroup, suggesting that they had similar attitudes toward IC/CC. Because different modes of classification can be generated via LCA (1-subgroup model, 2-subgroup model, 3-subgroup model, and so on) (11) (Table 2), the LCA was used to identify the optimal model containing the smallest number of subgroups necessary to adequately describe the associations among the choice of either IC or CC, the degree of details provided about these treatments, and presentation mode of the therapeutic outcomes (11) (Tables 1, 2). The optimal number of subgroups were generally determined using the Lo-Mendell-Rubin likelihood ratio test (LRT) (12), the adjusted Bayesian information criterion (adjusted BIC), and the likelihood ratio χ^2^. Lo-Mendell-Rubin likelihood ratio test (LRT) is a test for LCA, and if the models' *P* < 0.05, such models can be accepted. Concerning the fit measures (parsimony and goodness of fit), a model with fewer parameters (or subgroups), relatively lower adjusted BIC and Akaike Information Criterion (AIC) values are preferred. After the LCA for the negative and positive framing groups, a 3-subgroup model was preferred based on the fit measures. After the LCA for the negative and positive framing groups, the participants in both the negative and positive groups were combined, and all of their responses to the items were re-analyzed via LCA as a whole. Then, univariate and multiple logistic regression analyses using the stepwise procedure were applied using SPSS 19.0 (IBM, Armonk, NY, USA) to identify the significant demographic, health, and attitude predictors of the participant subgroup classifications (*P* < 0.05); to establish a perinatal decision-making model; and to discover the integrated influence and mutual relationships among the framing scenarios, the degree of detail provided about IC and CC, the presentation mode regarding the therapeutic outcomes, and various sociodemographic factors.

**Table 2:** Model fit statistics for the different subgroup models in the negative framing group, the positive framing group, and both groups.

**Frame**	**No. of**	**No. of**	**Log**	**AIC**	**BIC**	**Adj BIC**	**Likelihood**	**Df**	***P***	**Entropy**	**LRT**	**Delta**	***P***
**type**	**subgroups^a^**	**parameters^b^**	**likelihood**				**ratio χ^2^**					**df**	
**NEGATIVE^c^**													
	1	4	−443.194	894.388	908.737	896.055	244.154	10	0.0000	–	–	–	–
	2	9	−336.770	691.541	723.826	695.291	37.969	6	0.0000	0.933	205.491	5	0.0000
	3	14	−319.460	666.920	717.142	672.753	3.349	1	0.0673	0.877	33.424	5	0.0000
	4	19	−318.084	674.168	742.325	682.084	–	–	–	0.908	2.657	5	0.1100
**POSITIVE^c^**													
	1	4	−402.003	812.006	826.887	814.201	176.246	11	0.0000	–	–	–	–
	2	9	−332.802	683.605	717.087	688.544	37.845	6	0.0000	0.876	133.726	5	0.000
	3	14	−317.318	662.636	714.720	670.319	6.876	1	0.0087	0.977	29.922	5	0.000
	4	19	−314.187	666.374	737.060	676.801	–	–	–	0.881	6.050	5	0.0105
**TOTAL^d^**													
	1	5	−1,248.094	2,506.188	2,527.934	2,512.061	434.198	25	0.0000	–	–	–	–
	2	11	−1,071.153	2,164.306	2,212.146	2,177.226	88.540	20	0.0000	0.957	–	–	–
	3	17	−1,037.214	2,108.429	2,182.364	2,128.396	20.663	14	0.1106	0.985	–	–	–
	4	23	−1,033.117	2,112.235	2,212.265	2,139.250	12.470	8	0.1314	0.950	–	–	–

*AIC, Akaike information criterion; BIC, Bayesian information criterion; LRT, Lo-Mendell-Rubin likelihood ratio test*.

a *Different modes of classifications can be developed via LCA (1-subgroup model, 2-subgroup model, 3-subgroup model, and so on)*.

b *Concerning the fit measures (parsimony and goodness of fit), a model with fewer parameters (or subgroups), relatively lower BIC and AIC values and a significant p-value for LRT (< 0.05) is preferred*.

c *After the LCA for the negative and positive framing groups, a 3-subgroup model was preferred based on the fit measures*.

d *After the LCA for the negative and positive framing groups, the participants in both the negative and positive groups were combined, and all of their responses to the items were re-analyzed via LCA as a whole. According to the fit measures, the 3-subgroup model was optimal*.

## Results

### χ^2^-test of the endorsement rates for IC across different framing groups and items

A χ^2^-test was performed, and a framing effect with regard to Item 2 (the outcomes of IC and CC presented as large-base-specific figures after a brief description of the delivery, IC, and CC; see Table 1) was found (endorsement rate of IC in Neg vs. Pos: 87.7 vs. 94.6%; χ^2^ = 8.503, *P* = 0.004). Another χ^2^-test was applied to compare the endorsement rates associated with IC among different items in the Neg group (Table 3) and Pos group (Table 4). Regardless of the framing message, no significant differences were found in the endorsement rate of IC between Items 1 and 2 or in the endorsement rate of IC between Items 3 and 4, indicating that presentation mode did not influence preferences for IC (SF vs. LF). Moreover, significant differences were found in the endorsement rates for IC between Items 1 and 3 (SF; Neg: 90.7 vs. 81.4%; χ^2^ = 9.301, *P* = 0.002, see Table 3; Pos: 94.7 vs. 81.8%; χ^2^ = 24.125, *P* < 0.001, see Table 4) and between Items 2 and 4 (LF; Neg: 87.7 vs. 77.6%; χ^2^ = 9.327, *P* = 0.002, see Table 3; Pos: 94.6 vs. 83.7%; χ^2^ = 18.550, *P* < 0.001, see Table 4), demonstrating that IC endorsement rates decreased as the amount of information increased (from brief to detailed), regardless of the framing or presentation mode.

**Table 3:** χ^2^-test of the endorsement rates of IC across different items in the negative framing group.

**Information of background and treatment**		**Brief information**		**Detailed information**	
Item No.		1	2	3	4
Brief information	1	–	1.180 (*p* = 0.277)	9.301 (*p* = 0.002)	16.617 (*p* < 0.001)
	2		–	3.994 (*p* = 0.046)	9.327 (*p* = 0.002)
Detailed information	3			–	1.165 (*p* = 0.280)
	4				–

*α′ = 0.007*.

**Table 4:** χ^2^-test of the endorsement rates of IC across different items in the positive framing group.

**Information of background and treatment**		**Brief information**		**Detailed information**	
Item No.		1	2	3	4
Brief information	1	–			
	2	0.002 (*p* = 0.969)	–		
Detailed information	3	24.125 (*p* < 0.001)	23.564 (*p* < 0.001)	–	
	4	19.029 (*p* < 0.001)	18.550 (*p* < 0.001)	0.349 (*p* = 0.555)	–

*α′ = 0.007*.

### LCA of the endorsement rates of IC

**Figure 1:**
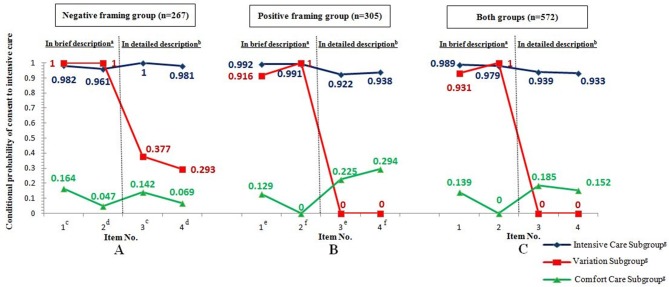
Conditional probability of consent to IC^h^ with regard to four items among the three subgroups of participants in the negative framing group **(A)**, positive framing group **(B)**, and both groups **(C)**. **a** The participants were presented with brief descriptions of a threatened delivery, IC, and CC before they completed Items 1 and 2. **b** The participants were presented with detailed descriptions of a threatened delivery, IC, and CC before they completed Items 3 and Item 4. **c** Item 1 in the negative framing group (followed by a brief description) was the same as Item 3 (followed by a detailed description), with the therapeutic outcomes of IC and CC presented as SF (100 infants). **d** Item 2 in the negative framing group (followed by a brief description) was the same as Item 4 (followed by a detailed description), with the therapeutic outcomes of IC and CC presented as LF (77,000 infants). **e** Item 1 in the positive framing group (followed by a brief description) was the same as Item 3 (followed by a detailed description), with the therapeutic outcomes of IC and CC presented as SF (100 infants). **f** Item 2 in positive framing group (followed by a brief description) was the same as Item 4 (followed by a detailed description), with therapeutic outcomes of IC and CC presented as LF (77,000 infants). **g** The subgroup classification was determined based on an LCA after the positive or negative framing was randomized and the 4 items were completed. **h** The conditional probability of consent to IC for each item was calculated via the LCA; a high conditional probability indicated that the participants had more favorable attitudes toward IC (e.g., the participants in the variation subgroup strongly opted for IC after a brief description but reversed their choice to CC after a detailed description, regardless of presentation mode or framing type.

The LCA performed for both the Neg and Pos groups indicated that a 3-subgroup model was optimal for both framing types, according to the LRT of the model's fit (12) (Neg: adjusted BIC = 672.753; LRT = 33.424, *P* < 0.0001; Likelihood Ratio χ^2^ = 3.349, *P* = 0.0673; Pos: adjusted BIC = 670.319; LRT = 29.922, *P* < 0.001; Likelihood Ratio χ^2^ = 6.876, *P* = 0.0087; Table 2). Therefore, the two groups were combined and set to obtain the same outcome expectations for LCA. This technique indicated an optimal 3-subgroup model for the overall calculations: when there were three subgroups, the adjusted BIC was the lowest value (2,128.396), and Likelihood Ratio χ^2^ is 20.663 (Df = 1, *P* = 0.1106), which meant the 3-subgroup model is optimal to describe the associations among the choice of treatment, the degree of details provided about these treatments, and presentation mode of the therapeutic outcomes (see Table 2). The LCA findings demonstrated that 488 (85.31%) of the 572 participants were classified into the IC subgroup (i.e., they showed a significant preference for IC), whereas 38 participants (6.65%) were classified into the CC subgroup (i.e., they showed a clear preference for CC). In addition, 46 participants (8.04%) were classified in the variation subgroup; these participants strongly opted for IC after an initial brief description but reversed their choice from IC to CC after being given a more detailed description, regardless of presentation mode or framing message (Figure 1). The classification probabilities for the most likely subgroup membership (i.e., latent class), the conditional probability, and subgroup probability were also calculated (Supplementary Tables S2, S3).

### Logistic regression analyses

Univariate analyses were performed for all of the expected variables associated with the latent classes. The variables nationality, occupation, participant type, check-up frequency, personality, and the importance of autonomy were excluded from the multivariate model (*P* > 0.05; Supplementary Table S4). However, some potentially important variables, such as parenthood, pregnancy, gender, age, religious affiliation, marital status, and the importance of quality of life were entered into the multiple regression model, regardless of significance.

Using backward stepwise selection, the multivariate analysis of the sociodemographic, health status and attitude predictors to classify the participants into subgroups showed that factors of Frame type, Parenthood, Education, Numeracy, and Importance of preservation of life were entered into the multivariable logistic regression model (*P* < 0.05, see Table 5). The IC subgroup was the reference subgroup. The multivariate models demonstrated that in contrast with the IC subgroup, 2.648 times more CC subgroup participants were placed in the Neg than the Pos group; 0.114 times more CC subgroup participants did not have children than those who did; 0.346 (or 0.240) times more CC subgroup participants had only a primary/junior middle school (or high school) education than those who had a college/postgraduate education; 3.937 times more CC subgroup participants demonstrated low numeracy than those who showed high numeracy; and 8.850 (or 4.373) times more CC subgroup participants made an argument against (or were uncertain about) the “importance of the preservation of life” than those who consented to the preservation. Moreover, in contrast to the IC subgroup, 3.686 (or 3.631) times more variation subgroup participants made an argument against (or were uncertain about) the “importance of the preservation of life” than those who consented (see Table 5). The multivariate model was established as follows:

**Table 5:** A multivariate analysis of the sociodemographic, health status and attitude predictors to classify the participants into subgroups.

**Variables**	**Category**	**Variation: IC**^**a**^				**CC: IC**^**a**^			
		**B**	**SE**	**OR (95% CIs)**^b^	***P***	**B**	**SE**	**OR (95% CIs)**^b^	***P***
Frame type	Neg	0.080	0.335	1.084 (0.562–2.089)	0.810	0.974	0.384	2.648 (1.246–5.625)	0.011
	Pos			1				1	
Parenthood	No	−1.825	1.035	0.161 (0.021–1.226)	0.078	−2.171	1.087	0.114 (0.014–0.959)	0.046
	Yes			1				1	
Education	Primary/Junior middle school	−0.472	0.512	0.624 (0.229–1.703)	0.357	−1.061	0.484	0.346 (0.134–0.894)	0.028
	High school	0.210	0.399	1.233 (0.565–2.694)	0.599	−1.425	0.466	0.240 (0.096–0.600)	0.002
	College/Postgraduate			1				1	
Numeracy	Low score (≤ 9)	−0.119	0.352	0.888 (0.445–1.769)	0.735	1.370	0.408	3.937 (1.770–8.758)	0.001
	High score (10–11)			1				1	
Importance of preservation of life	Disagree (Strongly disagree + disagree)	1.305	0.395	3.686 (1.698–8.001)	0.001	2.180	0.484	8.850 (3.427–22.854)	<0.001
	Uncertain	1.290	0.446	3.631 (1.516–8.697)	0.004	1.475	0.592	4.373 (1.371–13.942)	0.013
	Agree (Strongly agree + agree)			1				1	
Constant		−3.031	0.450		<0.001	−4.155	0.578		<0.001

*IC, intensive care subgroup; Variation, variation subgroup; CC, comfort care subgroup; B, unstandardized β coefficient; SE, standard error (β); OR, odds ratio; CIs, confidence intervals*.

a *The IC subgroup was the reference subgroup*.

b *Multiple logistic regression models were generated using backward stepwise selection*.

**Y**_IC_ = 0

**Y**_Variation_ = −3.031 + 1.305^*^Argument against the “importance of preservation of life” + 1.290^*^Uncertain viewpoint about the “importance of preservation of life”

**Y**_CC_ = −4.155 + 0.974^*^Negative frame – 2.171^*^No parenthood – 1.061^*^Primary/Junior middle school – 1.425^*^High school + 1.370^*^Low numeracy + 2.180^*^Argument against the “importance of preservation of life” + 1.475^*^Uncertain viewpoint about the “importance of preservation of life”

The intended probabilities of classification into different subgroups for each person could be calculated according to the following equations (e = 2.71828):

Probability of classification into IC = e^YIC^/[e^YIC^ + e^Yvariation^ + e^Ycc^]

Probability of classification into Variation = e^Yvariation^/[e^YIC^ + e^Yvariation^ + e^Ycc^]

Probability of classification into CC = e^YCC^/[e^YIC^ + e^Yvariation^ + e^Ycc^].

## Discussion

Consensus argues that the format of the presentation of medical outcomes (i.e., how they are “framed”) affects patient and clinician preferences for treatment and medical decisions (13). Our data indicate that framing messages and other factors influence participants' perinatal decisions. Item 1 and Item 3 had the same contents (the outcomes of IC and CC presented as small-base-specific figures; SF; Table 1), so did Item 2 and Item 4 (the outcomes of IC and CC presented as large-base-specific figures; LF; Table 1). Item 1 and Item 2 were presented after a brief description of the delivery, IC, and CC, while Item 3 and Item 4 were presented after a detailed description of the delivery, IC, and CC. The fact that framing effect was found only with regard to Item 2 and not the others 3 items demonstrated that there were mutual effects among faming effect, presentation mode, and background information. The absence of the framing effect in Item 1 and Item 3 indicated that small-base-specific figures can reduce the framing bias, regardless of the background information. Compared to the framing effect in Item 2, the absence of the framing effect in Item 4 implied that detailed background information about the IC and CC can also reduce the framing bias.

In addition, the perinatal decision model showed that in contrast to the IC subgroup, 2.648 times more CC subgroup participants were placed in the Neg vs. the Pos group, indicating that respondents for whom prognosis was framed as mortality data preferred CC, whereas respondents for whom prognosis was framed as survival data preferred IC. These findings support the hypothesis that a risk-seeking option is substantially more preferable than a risk-averse option in medical perinatal decision making when the therapeutic outcome is positively framed (1, 13, 14) and reverses the conventional framing effect in which the risk-averse option is generally more attractive when the therapeutic outcome is negatively framed (15, 16).

In contrast to a previous study (17), we did not observe any direct effects of presentation mode (SF vs. LF) on perinatal decision making. This null finding might be partly because of the repeated-measure issue or the limited presentation modes, suggesting that we should employ another subtle procedure and include various presentation modes (e.g., pictographs vs. bar graphs) in future studies (18). Although a repeated-measure design might make participants recall their first choice, simply tend to ignore the varying description, and remain consistent across items, our results still indicated that the endorsement rates of IC significantly decreased as the amount of information increased (from brief to detailed), regardless of the framing type or presentation mode. This reduction suggests that providing detailed information on delivery and treatment has powerful effects on perinatal decision making and corroborates our claim that the risk-averse option in perinatal medical decision is more attractive when described more fully. This finding has previously been supported in other settings (2, 4). A more detailed description of CC might seem more like a treatment than “giving up” (3), whereas a more detailed description of the prognosis of delivery would clearly highlight the risk of IC to participants; hence, their choice reverses from IC to CC. This attitude reversal toward medical care was also reflected in the variation subgroup. Participants in variation subgroup worrying more about future physical or mental disabilities were more likely to choose IC when presented with brief background information, but changed their initial choice when presented with a detailed description of therapeutic outcome (Figure 1). However, previous studies with different levels of detailed descriptions of the threatened delivery did not corroborate this manipulation of detail (3). Other possible reasons for this inconsistency include differences in patient identity, race, age, ethnicity, and culture as well as procedural designs. Thus, it is necessary to replicate our findings in other datasets.

In addition to design features, medical preferences might be affected by participant characteristics (19). According to our multivariate models, negative framing, higher education, parenthood, and poor numeracy predicted preferences for CC (in the CC but not IC subgroups). Numeracy (the ability to understand and use quantitative information) is an important variable. During medical communication, clinicians often convey information about treatment and prognosis to patients, and much of this information is described as numbers (20). In many cases, numerical information is difficult for both patients and physicians to understand, especially those whose numeracy is too poor to process important health messages and medical information (21). For example, low numeracy is related to the tendency to overestimate one's own cancer risk, and this overestimation affects the perception of the benefits of cancer screening and screening behaviors (22, 23). Therefore, participants with poor numeracy might overestimate the risk of active treatment of premature infants and therefore be more likely to opt for CC. In addition, the effect of education and parenthood has seldom been reported with regard to perinatal decision making. Speculatively, highly educated parents with children might worry more about the physical or mental disabilities of premature infants and therefore be more likely to choose CC. Because higher education is generally associated with better numeracy, our findings showing that both higher education and poor numeracy were associated with the individual preferences for CC seem paradoxical. In fact, higher education does not ensure higher grade-level skills, which is particularly true for mathematics (24). Furthermore, literate, educated people can also have trouble understanding important numerical concepts (25). In other words, years of education cannot be assumed to translate into numeracy skill (21, 26). Previous studies have reported that ~60% of participants choose IC (1, 3), and our study showed that the majority of participants (85.31%) preferred IC (IC subgroup) and tended to highly value the preservation of life. This finding suggests that the value of preserving life was culturally universal across all conditions.

In summary, our data reveal that both external influences (message framing and detailed descriptions) and internal influences (demographics and value predictors) simultaneously contribute to the perinatal decision making of our participants. Notably, most decision-making studies concerning medical and health policy have been conducted in Europe or North America. Because the current study was conducted in Beijing, it implicitly addressed the question of how cultural factors (e.g., religious beliefs), which vary widely across continents, affect these results. This study also addressed how much these factors are affected by a common set of psychological variables that are largely independent of culture and are just part of the human cognitive machinery (e.g., cognitive biases). Alternatively, this value might be culturally universal. We are encouraged to continue to address this issue. To generalize our results, our current novel findings require replication using other samples and under varying circumstances.

### Limitations

There are some important aspects not evaluated in the present study. Most importantly, it should be discussed that during the counseling before birth, obstetrics and neonatologists should explain to the parents their own institutional approach to the “Extremely premature birth,” declaring the incidence of survival and survival without neurodevelopmental impairment of extremely preterm infants. Moreover, it should be taken into account the factors potentially affecting the clinical outcomes: (1) non-modifiable risk factors (e.g., race, sex, birth weight, and gestational age); (2) modifiable obstetrics practices (antenatal interventions, such as steroids, antibiotics, MgSO_4_ for neuroprotection; site and mode of delivery, delayed cord clamping etc.); (3) modifiable neonatal practices (initial resuscitation in the delivery room and subsequent care). (4) Written consent vs. verbal consent: in the present study, we only represent the participants with written information, and fail to discuss the influence of communication styles (written consent vs. verbal consent) upon perinatal decision making; clinically, the latter is employed more popularly. In summary, careful and punctual assessment on clinical indications is a fundamental factor that can also influence perinatal decision making, which should be involved in the future research. Clinical indications should be the first step on which developing any further consideration about newborn's best interest. Additionally, more suitable participants should be chosen as the targeted population to safeguard the homogeneity in the future study.

## Conclusions

Our data revealed that participants prefer risk seeking (e.g., IC) under conditions of positive framing rather than negative framing. Of the participants who initially preferred IC, the majority (85.31%) had well-articulated preferences that were unchanged by external intervention; however, a minority (8.04%) changed their initial choice to CC after detailed descriptions of the delivery, IC, and CC were presented. The remaining participants (6.65%) showed intrinsic preferences for CC that were unaffected by detail manipulation but were affected by the framing effect and various sociodemographic variables.

In summary, many internal (demographic and value predictors) and external factors (message framing and detailed descriptions) affect perinatal decision making. Significant effort should be made to design unbiased communication between doctors and family members to ensure an effective comprehension of risks and benefits of treatments for extremely premature infants. More detailed background information, improved understandability of numerical information (21), and a neutral tone of voice when presenting the possible treatments (11) might be of great help to family members when making unbiased perinatal decisions.

## Author contributions

JG and YH designed the study and performed the experiments. HL, YZ, and JG designed the study and analyzed the data. JG and WX searched the literature, collected the data and wrote the manuscript. HG, WW, WZ, LX, LD, and JL performed the experiments and collected and interpreted the data. YZ prepared the psychological assessment, materials, analysis tools and revised the manuscript. YH supervised the study.

### Conflict of interest statement

The authors declare that the research was conducted in the absence of any commercial or financial relationships that could be construed as a potential conflict of interest.
