# Supplementary material for: How to Best Convey Information About Intensive/Comfort Care to the Family Members of Premature Infants to Enable Unbiased Perinatal Decisions
Source: Front Pediatr. 2018 Nov 16;6:348. doi: 10.3389/fped.2018.00348 (PMC6251209; doi:10.3389/fped.2018.00348)
Supplement: Supplementary file 1 [file Table_1.DOCX]

**Supplemental Digital Content-Tables**

**S1 Table Socio-demographic, health statue / knowledge, psychological characteristics, and attitudes data of the participants in the negtive framing group and the positive framing group**

| Variables | Negative  (n=267) | | | Positive  (n=305) | *P* |
| --- | --- | --- | --- | --- | --- |
| *Socio-demographic Characteristics* | | | |  |  |
| Subject types | |  | |  | 0.232 |
| Inpatients | | 147 | | 183 |  |
| Inpatients’ relatives | | 120 | | 122 |  |
| Gender, *n* (%) | |  | |  | 0.084 |
| Male | | 153 | | 156 |  |
| Female | | 105 | | 144 |  |
| Education | |  | |  | 0.696 |
| Primary / Junior middle school | | 71 | | 72 |  |
| Hight school | | 109 | | 129 |  |
| College/ Postgraduate | | 70 | | 85 |  |
| Age | |  | |  | 0.291 |
| 18–39 y | | 63 | | 58 |  |
| 40–59 y | | 109 | | 128 |  |
| >60 y | | 83 | | 110 |  |
| Nationality | |  | |  | 0.205 |
| Han | | 253 | | 280 |  |
| others | | 14 | | 24 |  |
| Marital status | |  | |  | 0.733 |
| married/living with partner | | 230 | | 265 |  |
| unmarried/divorced/widowed | | 35 | | 37 |  |
| Religious belief | |  | |  | 0.341 |
| with religious belief | | 29 | | 26 |  |
| Without religious belief | | 235 | | 276 |  |
| Occupation | |  | |  | 0.129 |
| [manual worker](http://www.iciba.com/manual_workers) | | 156 | | 156 |  |
| mental labourer | | 84 | | 111 |  |
| Numeracy | |  | |  | 0.729 |
| Low score(<10) | | 115 | | 127 |  |
| High score (10-11) | | 152 | | 178 |  |
| *Health statue and knowledge/attention* | | | |  |  |
| Check-up freqency (including hospitalization) | | | |  | 0.713 |
| <once 6months | 219 | | | 245 |  |
| once 6 months | 25 | | | 35 |  |
| > once 6 months | 21 | | | 23 |  |
| Health self-rating |  | | |  | **0.041** |
| very poor+poor | 46 | | | 64 |  |
| Intermediate | 157 | | | 192 |  |
| good+best | 64 | | | 48 |  |
| Focus on health |  | | |  | 0.520 |
| not at all + less | 76 | | | 79 |  |
| Intermediate | 120 | | | 132 |  |
| more + extremely | 67 | | | 89 |  |
| *Psychological characteristics and attitudes* | | | |  |  |
| Personality | | |  |  | 0.316 |
| Introverted | | | 125 | 160 |  |
| Extroverted | | | 120 | 129 |  |
| Importance of quality of life | | |  |  | 0.111 |
| Strongly disagree + disagree | | | 20 | 37 |  |
| Uncertain | | | 25 | 34 |  |
| Stronly agree + agree | | | 220 | 230 |  |
| Importance of autonomy | | |  |  | 0.560 |
| Strongly disagree + disagree | | | 61 | 68 |  |
| Uncertain | | | 87 | 89 |  |
| Stronly agree + agree | | | 116 | 146 |  |
| Importance of preservation of life | | |  |  | 0.645 |
| Strongly disagree + disagree | | | 85 | 88 |  |
| Uncertain | | | 42 | 55 |  |
| Stronly agree + agree | | | 139 | 161 |  |
| Parenthood | | |  |  | 0.736 |
| No | | | 24 | 25 |  |
| Yes | | | 243 | 280 |  |
| Knowing parents with premature children | | | |  | 0.886 |
| No | | | 189 | 215 |  |
| Yes | | | 71 | 83 |  |
| SCL-90-R | | |  |  |  |
| Total average | | | 1.45 ±0.48 | 1.50 ±0.47 | 0.233 |
| Number of positive items | | | 25.10 ±22.31 | 28.10 ±21.85 | 0.126 |
| Number of negtitive items | | | 64.90 ±22.31 | 61.90 ±21.85 | 0.126 |
| Average of postive items | | | 2.44 ±0.38 | 2.46 ±0.42 | 0.598 |
| Somatisation | | | 1.57 ±0.53 | 1.62 ±0.54 | 0.334 |
| Obsessive-compulsive | | | 1.61 ±0.63 | 1.67 ±0.63 | 0.297 |
| Interpersonal sensitivity | | | 1.43 ±0.53 | 1.49 ±0.54 | 0.173 |
| Depression | | | 1.45 ±0.59 | 1.50 ±0.54 | 0.305 |
| Anxiety | | | 1.36 ±0.51 | 1.42 ±0.49 | 0.172 |
| Hostility | | | 1.47 ±0.54 | 1.57 ±0.62 | 0.052 |
| Phobic anxiety | | | 1.33 ±0.53 | 1.35 ±0.51 | 0.770 |
| Paranoid ideation | | | 1.36 ±0.50 | 1.41 ±0.55 | 0.318 |
| Psychoticism | | | 1.35 ±0.49 | 1.40 ±0.49 | 0.320 |
| Additional factors | | | 1.58 ±0.61 | 1.59 ±0.56 | 0.815 |

**S2 Table Classification probabilities for the most likely subgroup (latent class) membership in the negative framing group, the positive framing group, and both groups (total)**

| Frame type |  | Classification Probabilities for the Most Likely Subgroup Membership | | |
| --- | --- | --- | --- | --- |
|  | **Subgroup** | **1** | **2** | **3** |
| Negative |  |  |  |  |
|  | **1** | **0.979** | 0.017 | 0.003 |
|  | **2** | 0.122 | **0.867** | 0.011 |
|  | **3** | 0.006 | 0.008 | **0.985** |
| Positive |  |  |  |  |
|  | **1** | **0.994** | 0.005 | 0.002 |
|  | **2** | 0.006 | **0.994** | 0.000 |
|  | **3** | 0.009 | 0.006 | **0.985** |
| Total |  |  |  |  |
|  | **1** | **0.949** | 0.044 | 0.007 |
|  | **2** | 0.012 | **0.951** | 0.037 |
|  | **3** | 0.004 | 0.001 | **0.995** |

**S3 Table Conditional probability of intensive care in the negative framing group, the positive framing group, and both groups**

| Information of background and treatment | Item No. |  | | Negative frame group | | | |  | | Positive frame group | | | |  | | Both groups (Total) | | |
| --- | --- | --- | --- | --- | --- | --- | --- | --- | --- | --- | --- | --- | --- | --- | --- | --- | --- | --- |
|  |  | **Subgroup**  **(Latent class)** | **1** | | **2** | **3** |  | | **1** | | **2** | **3** |  | | **1** | | **2** | **3** |
| Brief information |  |  |  | |  |  |  | |  | |  |  |  | |  | |  |  |
|  | 1 |  | 0.982 | | 1.000 | 0.164 |  | | 0.992 | | 0.916 | 0.129 |  | | 0.989 | | 0.931 | 0.139 |
|  | 2 |  | 0.961 | | 1.000 | 0.047 |  | | 0.991 | | 1.000 | 0.000 |  | | 0.979 | | 1.000 | 0.000 |
| Detailed information |  |  |  | |  |  |  | |  | |  |  |  | |  | |  |  |
|  | 3 |  | 1.000 | | 0.377 | 0.142 |  | | 0.922 | | 0.000 | 0.225 |  | | 0.939 | | 0.000 | 0.185 |
|  | 4 |  | 0.981 | | 0.293 | 0.069 |  | | 0.938 | | 0.000 | 0.294 |  | | 0.933 | | 0.000 | 0.152 |
| Subgroup probability(%) |  |  | 73.6 | | 16.6 | 9.8 |  | | 87.7 | | 7.8 | 4.5 |  | | 85.31 | | 8.04 | 6.65 |

**S4 Table Univariate analysis of sociodemographic, health status and attitude predictors for the classification of participants into subgroups**

| Variables | Category | IC  (n=488) | Variation  (n=46) | CC  (n=38) |  | OR(95% CI) | *P* |
| --- | --- | --- | --- | --- | --- | --- | --- |
|  |  | **n(%)** | **n(%)** | **n(%)** | **IC as a reference^a^** | |  |
| Frame type | Negative | 221(45.3) | 21(45.7) | 25(65.8) | *2:1****^b^*** | 1.015(0.553-1.862) | 0.962 |
|  | Positive | 267(54.7) | 25(54.3) | 13(34.2) | *3:1****^c^*** | 2.323(1.161-4.648) | **0.017** |
| Gender | Male | 261(54.6) | 23(53.5) | 25(67.6) | *2:1* | 0.956(0.511-1.788) | 0.888 |
|  | Female | 217(45.4) | 20(46.5) | 12(32.4) | *3:1* | 1.732(0.850-3.528) | 0.130 |
| Education | Primary/Junior middle school | 127(27.7) | 7(17.1) | 9(24.3) | *2:1* | 0.574(0.219-1.506) | 0.259 |
|  | Hight school | 206(45.0) | 22(53.7) | 10(27.0) | *2:1* | 1.112(0.532-2.326) | 0.777 |
|  | College/ Postgraduate | 125(27.3) | 12(29.3) | 18(48.6) | *3:1* | 0.492(0.213-1.137) | 0.097 |
|  |  |  |  |  | *3:1* | 0.337(0.151-0.754) | **0.008** |
| Age | 18–39 y | 109(23.1) | 8(18.6) | 4(10.8) | *2:1* | 0.788(0.323-1.922) | 0.600 |
|  | 40–59 y | 201(42.7) | 20(46.5) | 16(43.2) | *2:1* | 1.068(0.530-2.152) | 0.854 |
|  | >60 y | 161(34.2) | 15(34.9) | 17(45.9) | *3:1* | 0.348(0.114-1.061) | 0.063 |
|  |  |  |  |  | *3:1* | 0.754(0.369-1.539) | 0.557 |
| Nationality | Han | 453(93.0) | 45(97.8) | 35(92.1) | *2:1* | 3.377(0.452-25.259) | 0.236 |
|  | others | 34(7.0) | 1(2.2) | 3(7.9) | *3:1* | 0.876(0.256-2.994) | 0.832 |
| Marital status | unmarried/divorced/widowed | 64(13.3) | 4(8.7) | 4(10.5) | *2:1* | 0.624(0.216-1.797) | 0.382 |
|  | married/living with partner | 419(86.7) | 42(91.3) | 34(89.5) | *3:1* | 0.770(0.264-2.243) | 0.632 |
| Religious belief | with religious belief | 49(10.1) | 3(6.7) | 3(8.1) | *2:1* | 0.634(0.189-2.122) | 0.460 |
|  | Without religious belief | 435(89.9) | 42(93.3) | 34(91.9) | *3:1* | 0.783(0.232-2.645) | 0.694 |
| Occupation | [manual worker](http://www.iciba.com/manual_workers) | 275(63.2) | 22(55.0) | 15(46.9) | *2:1* | 0.711(0.370-1.366) | 0.306 |
|  | mental labourer | 160(36.8) | 18(45.0) | 17(53.1) | *3:1* | 0.513(0.250-1.056) | 0.070 |
| Subject types | Carer | 211(43.2) | 18(39.1) | 13(34.2) | *2:1* | 0.844(0.455-1.567) | 0.591 |
|  | Inpatients | 277(56.8) | 28(60.9) | 25(65.8) | *3:1* | 0.683(0.341-1.366) | 0.281 |
| Numeracy | Low score(≤ 9) | 200(41.0) | 17(37.0) | 25(65.8) | *2:1* | 0.844(0.452-1.578) | 0.595 |
|  | High score (10-11) | 288(59.0) | 29(63.0) | 13(34.2) | *3:1* | 2.769(1.383-5.543) | **0.004** |
| Check-up freqency | <once 6months | 393(81.2) | 43(93.5) | 28(73.7) | *2:1* | 4.158(0.557-31.043) | 0.165 |
|  | once 6 months | 53(11.0) | 2(4.3) | 5(13.2) | *2:1* | 1.434(0.125-16.392) | 0.772 |
|  | > once 6 months | 38(7.9) | 1(2.2) | 5(13.2) | *3:1* | 0.541(0.198-1.484) | 0.233 |
|  |  |  |  |  | *3:1* | 0.717(0.194-2.651) | 0.618 |
| Health self-rating | Poor (very poor+poor) | 85(17.5) | 12(26.1) | 13(34.2) | *2:1* | 1.369(0.563-3.329) | 0.488 |
|  | Intermediate | 305(62.6) | 24(52.2) | 20(52.6) | *2:1* | 0.763(0.353-1.652) | 0.493 |
|  | Good (good+best) | 97(19.9) | 10(21.7) | 5(13.2) | *3:1* | 2.967(1.016-8.665) | **0.047** |
|  |  |  |  |  | *3:1* | 1.272(0.465-3.480) | 0.639 |
| Focus on health | Less (not at all + less) | 129(26.8) | 9(20.9) | 17(44.7) | *2:1* | 0.762(0.310-1.869) | 0.552 |
|  | Intermediate | 222(46.1) | 22(51.2) | 8(21.1) | *2:1* | 1.082(0.518-2.258) | 0.834 |
|  | More (more + extremely) | 131(27.2) | 612(27.9) | 13(34.2) | *3:1* | 1.328(0.620-2.845) | 0.466 |
|  |  |  |  |  | *3:1* | 0.363(0.147-0.899) | **0.029** |
| Personality | Introverted | 246(53.9) | 25(56.8) | 14(41.2) | *2:1* | 1.123(0.602-2.097) | 0.715 |
|  | Extroverted | 210(46.1) | 19(43.2) | 20(58.8) | *3:1* | 0.598(0.295-1.212) | 0.154 |
| Importance of  quality of life | Disagree (Strongly disagree + disagree) | 49(10.1) | 3(6.8) | 5(13.2) | *2:1* | 0.632(0.188-2.127) | 0.459 |
|  | Uncertain | 53(11.0) | 4(9.1) | 2(5.3) | *2:1* | 0.779(0.267-2.274) | 0.648 |
|  | Agree (Strongly agree + agree) | 382(78.9) | 37(84.1) | 31(81.6) | *3:1* | 1.257(0.467-3.385) | 0.650 |
|  |  |  |  |  | *3:1* | 0.465(0.108-1.999) | 0.303 |
| Importance of autonomy | Disagree (Strongly disagree + disagree) | 103(21.3) | 16(34.8) | 10(26.3) | *2:1* | 1.687(0.845-3.365) | 0.138 |
|  | Uncertain | 152(31.5) | 9(19.6) | 15(39.5) | *2:1* | 0.643(0.287-1.441) | 0.283 |
|  | Agree (Strongly agree + agree) | 228(47.2) | 21(45.7) | 13(34.2) | *3:1* | 1.703(0.723-4.010) | 0.223 |
|  |  |  |  |  | *3:1* | 1.731(0.801-3.740) | 0.163 |
| Importance of preservation of life | Disagree (Strongly disagree + disagree) | 130(26.7) | 19(41.3) | 24(63.2) | *2:1* | 2.709(1.334-5.500) | **0.006** |
|  | Uncertain | 78(16.0) | 12(26.1) | 7(18.4) | *2:1* | 2.851(1.282-6.343) | **0.010** |
|  | Agree (Strongly agree + agree) | 278(57.3) | 15(32.6) | 7(18.4) | *3:1* | 7.332(3.080-17.453) | **<0.001** |
|  |  |  |  |  | *3:1* | 3.564(1.214-10.467) | **0.021** |
| Parenthood | No | 46(9.4) | 2(4.3) | 1(2.6) | *2:1* | 0.437(0.103-1.860) | 0.263 |
|  | Yes | 442(90.6) | 44(95.7) | 37(97.4) | *3:1* | 0.260(0.035-1.937) | 0.188 |
| Knowing parents with premature infants | No | 352(73.5) | 31(73.8) | 21(56.8) | *2:1* | 1.017(0.496-2.083) | 0.964 |
|  | Yes | 127(26.5) | 11(26.2) | 16(43.2) | *3:1* | 0.474(0.240-0.936) | **0.032** |

**Abbreviations:** IC-Intensive care subgroup; Variation-Variation subgroup; CC-Comfort care subgroup; OR=Odds Ratios; CI= Confidence Interval

a Univariable odds ratios were calculated with simple logistic regression, and Intensive care subgroup acted as the reference;

b 2:1= Variation subgroup: Intensive care subgroup;

c 3:1= Comfort care subgroup: Intensive care subgroup;
