# Supplementary material for: How to Best Convey Information About Intensive/Comfort Care to the Family Members of Premature Infants to Enable Unbiased Perinatal Decisions
Source: Front Pediatr. 2018 Nov 16;6:348. doi: 10.3389/fped.2018.00348 (PMC6251209; doi:10.3389/fped.2018.00348)
Supplement: Supplementary file 2 [file Data_Sheet_1.DOCX]

**Supplemental Digital Content-Appendices**

**Number of Appendices included in the Supplement:** 6

**S1 Appendix** *Brief and detailed descriptions of threatened delivery, intensive care and comfort care*

The brief and detailed descriptions of threatened delivery, intensive care and comfort care were referred to the study of Haward et al.[^2^](#_ENREF_2)

**S2 Appendix** *Instructions*

Participants received a letter indicating that they would respond to a series of questions, which they were urged to answer carefully and truthfully as all choices were acceptable and rational.

**S3 Appendix** *Numeracy measure*

Participants were required to complete the Numeracy Scale[^1^](#_ENREF_1) which consisted of 3 general numeracy scale items and 8 expanded numeracy scale items.

**S4 Appendix** *Socio-demographic, health & attitude questionnaire*

The questionnaire contained not only socio-demographic questions (age, gender, nationality, marital status, et al.) but also participants’ health status.

**S5 Appendix** *Questionnaires of personal attitudes*

Questionnaires of personal attitudes were referred to the study of Haward et al.[^2^](#_ENREF_2)

**S6 Appendix** *Psychological characteristics*

**S1 Appendix**

**A brief description:**

If a baby was born very prematurely (23 weeks, for example), he or she would be at higher risk of severe developmental handicaps, and even unable to survive. For such kind of premature babies, two types of medical care are provided in the hospital, and their parents have to make their choices:

**A detailed description:**

A baby who is born in less than 37 weeks of gestation is premature. As for premature babies with over 28 weeks of gestation, they have a higher survival rate and probability of normal development. But if premature babies are born in less than 25 weeks, they will be at high risk for a variety of severe dysplasia, such as mental retardation, cerebral palsy, physical disability, deafness or blindness.

If a baby was born very prematurely (23 weeks, for example), he or she would be at higher risk of severe developmental handicaps, and even unable to survive. For such kind of premature babies, two types of medical care are provided in the hospital, and their parents have to make their choices:

Intensive care, which means that the baby will be transferred to the intensive care unit, and their breathing and heart beating will be supported and assisted by artificial breathing machine and other machines so as to make them try to stay alive.

Comfort care, which means that the babies will be transferred to a comfortable and warm places without any pain until they die a natural death. In the process, the doctors and nurses will take good care of the babies, and their parents can hold them.

**S2 Appendix**

**Instruction**

**Dear friends:**

We aim to popularize the common knowledge related to health, collect the general public’s current health status, and learn about people’s understanding of and preference for common treatment in medical decision making.

The findings of our study would help further improve health education, eliminate misunderstanding in health and health care, and promote healthy behaviours to safeguard everyone’s health to the greatest extent possible!

You are not only a participant but also a future beneficiary!

**Note:** Please fill in the answers in order and please do not review or modify your answers. No matter what you choose, your choices are accepted and appreciated, as there is no right or wrong answer!

We hope you will carefully and truthfully answer the questions as soon as possible. We will ensure that the detailed contents of personal information are kept confidential. Thank you for your support and cooperation!

**S3 Appendix**

**Here is a short quiz. Please try to complete the following questions:**

How many times would a coin land heads-up when tossed 1000 times?

**Answer:** (this is a practice question, and the answer will be provided to the participants)

1. Imagine that we roll a fair, six-sided die 1,000 times. Out of the 1,000 rolls, how many times do you think the die would come up even (2, 4, or 6)? ___

2. In a public welfare lottery, the chance of winning a ¥10·00 prize is 1%. What is your best guess of the number of people winning a ¥10·00 prize if 1,000 people each buy a single ticket for the public welfare lottery? ___

3. In a lottery draw at a supermarket, the chance of winning an [electromobile](http://www.iciba.com/electrombile) is 1 in 1,000. What percentage of the tickets in the lottery draw will win a [electromobile](http://www.iciba.com/electrombile)? ___

4. Which of the following numbers represents the greatest risk of getting a disease?

___ 1 in 100; ___ 1 in 1000; ___ 1 in 10 (put a tick” √” next to the answer )

5. Which of the following numbers represents the greatest risk of getting a disease?

___ 1%; ___ 10%; ___ 5% (put a tick” √” next to the answer )

6. If Person A’s risk of having an infarction is 1% in ten years, and person B’s risk is double that of A’s, what is B’s risk in ten years? ___

7. If Person A’s risk of having an infarction is 1 in 100 in ten years, and person B’s risk is double that of A’s, what is B’s risk in ten years? ___

8. If the chance of having an infarction is 10% in a population, how many people would be expected to get the disease:

A. Out of 100? ___

B. Out of 1000? ___

9. If the chance of having an infarction is 20 out of 100, this would be the same as having a ___% chance of getting the disease.

10. The chance of getting a viral infection is .0005. Out of 10,000 people, about how many of them are expected to become infected? ___

**S4 Appendix**

**Anonymous Demographic and Health Status Questionnaire**

**Tips:** Please fill in the answer directly on the line or place a tick “√” in the brackets next to the answer you would like to choose.

**1. Age:** ____years; **2. Gender:** _____; **3. nationality:** _____

**4. Present and past occupation:** __________________ (please fill in on the line)

**5. Education background:** ________(such as primary school, high school, college, Master’s or Doctoral degree).

**6. You might be characterized as:** Introverted ( ) or Extroverted ( )

**7. Religion or Belief:** None ( ); Yes ( );

if yes, please specify:________________________

**8. Marital status:**

① Single ( ) ② Boyfriend or Girlfriend ( ) ③ Married ( ) ④ Divorced ( ) ⑤ Widowed ( )

**9. What is your identity?**

① inpatient ( ) ② immediate family members of inpatients ( )

**10. How often you usually see a physician (including hospitalization):**

① less than once a year ( ) ② once a year ( ) ③ once / 6 months ( ) ④ 1-2 times / 3 months( ) ⑤ 1-2 times / month ( )

**11. Overall assessment of your current health:**

① very poor ( ) ② poor ( ) ③ intermediate ( ) ④ good ( ) ⑤ best ( )

**12. How well do you pay attention to your health?**

① Not at all ( ) ② less ( ) ③ intermediate ( ) ④ more ( ) ⑤ a great deal ( )

**S5 Appendix**

**1. Do you have children?** Yes ( ); No ( );

if yes, for how many children do you have? _______

**2.** **Do you have any friends or relatives who have had a premature infant?**

Yes ( ); No ( );

**3. Do you agree with the following viewpoints?**

**1) when making decision whether a premature baby should be placed in intensive care, Future physical or mental disabilities should not be considered.**

① strongly disagree ( ) ② disagree ( ) ③ uncertain ( )

④ agree ( ) ⑤ strongly agree ( )

**2) One’s quality of life is more important than his or her lifespan.**

① strongly disagree ( ) ② disagree ( ) ③ uncertain ( )

④ agree ( ) ⑤ strongly agree ( )

**3) No matter what happens, one shouldn’t tell others what they have to do.**

① strongly disagree ( ) ② disagree ( ) ③ uncertain ( )

④ agree ( ) ⑤ strongly agree ( )

**S6 Appendix**

The SCL-90-R[^3^](#_ENREF_3) was used to measure the psychological state of participants. It is a validated and reliable self-rated scale which is usually chosen to assess 10 postulated factors: depression, somatisation, anxiety, phobic anxiety, obsessive-compulsive, interpersonal sensitivity, hostility, psychoticism, paranoid ideation, and additional factors (sleep and concentration difficulties). The inventory comprised 90 items with a rating scale with 5 degrees of severity (from “not at all” to “extremely”) according to his or her mood state over the past week. We used the Chinese translation by Wang[^4^](#_ENREF_4).

**eReferences**

**1.** Lipkus IM, Samsa G, Rimer BK. General performance on a numeracy scale among highly educated samples. *Medical decision making : an international journal of the Society for Medical Decision Making.* Jan-Feb 2001;21(1):37-44.

**2.** Haward MF, Murphy RO, Lorenz JM. Message framing and perinatal decisions. *Pediatrics.* Jul 2008;122(1):109-118.

**3.** Jalenques I, Galland F, Malet L, et al. Quality of life in adults with Gilles de la Tourette Syndrome. *BMC psychiatry.* 2012;12:109.

**4.** ZY. W. Self-reporting Inventory (SCL-90). *Shanghai Archives of Psychiatry.* 1984(02):68-70.
